# Phylogeography of the Tropical Planktonic Foraminifera Lineage *Globigerinella* Reveals Isolation Inconsistent with Passive Dispersal by Ocean Currents

**DOI:** 10.1371/journal.pone.0092148

**Published:** 2014-03-24

**Authors:** Agnes K. M. Weiner, Manuel F. G. Weinkauf, Atsushi Kurasawa, Kate F. Darling, Michal Kucera, Guido W. Grimm

**Affiliations:** 1 MARUM Center for Marine Environmental Sciences, University of Bremen, Bremen, Germany; 2 Institute of Biogeosciences, Japanese Agency for Marine Earth Science and Technology, Yokosuka, Japan; 3 School of Geosciences and Institute of Evolutionary Biology, University of Edinburgh, Edinburgh, United Kingdom; 4 Department of Palaeobiology, Swedish Museum of Natural History, Stockholm, Sweden; Institute of Biochemistry and Biology, Germany

## Abstract

Morphologically defined species of marine plankton often harbor a considerable level of cryptic diversity. Since many morphospecies show cosmopolitan distribution, an understanding of biogeographic and evolutionary processes at the level of genetic diversity requires global sampling. We use a database of 387 single-specimen sequences of the SSU rDNA of the planktonic foraminifera *Globigerinella* as a model to assess the biogeographic and phylogenetic distributions of cryptic diversity in marine microplankton on a global scale. Our data confirm the existence of multiple, well isolated genetic lineages. An analysis of their abundance and distribution indicates that our sampling is likely to approximate the actual total diversity. Unexpectedly, we observe an uneven allocation of cryptic diversity among the phylogenetic lineages. We show that this pattern is neither an artifact of sampling intensity nor a function of lineage age. Instead, we argue that it reflects an ongoing speciation process in one of the three major lineages. Surprisingly, four of the six genetic types in the hyperdiverse lineage are biogeographically restricted to the Indopacific. Their mutual co-occurrence and their hierarchical phylogenetic structure provide no evidence for an origin through sudden habitat fragmentation and their limitation to the Indopacific challenges the view of a global gene flow within the warm-water provinces. This phenomenon shows that passive dispersal is not sufficient to describe the distribution of plankton diversity. Rather, these organisms show differentiated distribution patterns shaped by species interactions and reflecting phylogenetic contingency with unique histories of diversification rates.

## Introduction

In many groups of marine microplankton, morphologically defined species tend to underestimate diversity [1], [2]. Cryptic speciation is prevalent in these groups, manifested in genetic differences that are not accompanied by the development of morphologically divergent traits [3]. In consequence, diversity patterns and species biogeography derived from observations of morphospecies may not reflect processes at the level of biological species.

This observation has consequences for the interpretation of biogeographic patterns of marine microplankton. At the morphological level, species often appear globally distributed, but their constituent cryptic lineages may show more differentiated patterns [4]. In theory, such spatially structured distribution may reflect either dispersal limitation, differential adaptation or niche incumbency [5], [6]. The fundamental difference among these scenarios lies in the ubiquity of gene flow and in the importance of species interactions. Under dispersal limitation, genetic drift associated with the establishment of abiotic barriers may lead to the differentiation of allopatric sister lineages. If dispersal is not the primary restriction and species interaction is of subdued importance, then distribution of species should reflect the spatial realization of suitable niches. If, however, species interactions are important then the occupancy of the realized niches will be influenced by competitive exclusion, leading to a pattern of niche incumbency. Because of the manifest differences among the predictions of these three scenarios, an observed species biogeography could in theory be used to draw conclusions about the importance of dispersal and species interactions for the distribution and diversity of marine plankton.

Because of the prevalence of cryptic speciation and the often cosmopolitan distribution of morphospecies in plankton, an assessment of these three end-member scenarios for biogeographic patterns requires an extensive global sampling of genetic diversity, covering the entire range of the studied lineage. Here we use the genetically most diverse morphospecies of planktonic foraminifera as a model to assess global biogeography of DNA-delineated cryptic species in view of these scenarios. Most morphospecies of planktonic foraminifera have a cosmopolitan distribution within their preferred temperature range [7] and evidence exists that gene flow in these obligate sexual outbreeders occurs on a global scale [8], [9]. On the other hand, there is abundant evidence that morphospecies of planktonic foraminifera represent complexes of reproductively isolated but morphologically indistinguishable cryptic species [4]. In most cases such cryptic species reveal restricted distribution patterns, indicating that biogeographies of morphospecies in this group are not representative for processes at the level of biological species [10]–[12].

Earlier studies of the phylogeography of planktonic foraminifera attempted to identify the pattern of speciation that has led to the observed distribution or the environmental factors influencing it, but the importance of biological interactions has been largely overlooked [4], [13], [14]. Aurahs *et al.* [10] first noted that the distribution of genetic lineages of *Globigerinoides ruber* could be best explained by competitive exclusion and the concept was then used by Seears *et al.* [15] to explain the occurrence of genetic types of planktonic foraminifera in the Arabian Sea.

**Figure 1:**
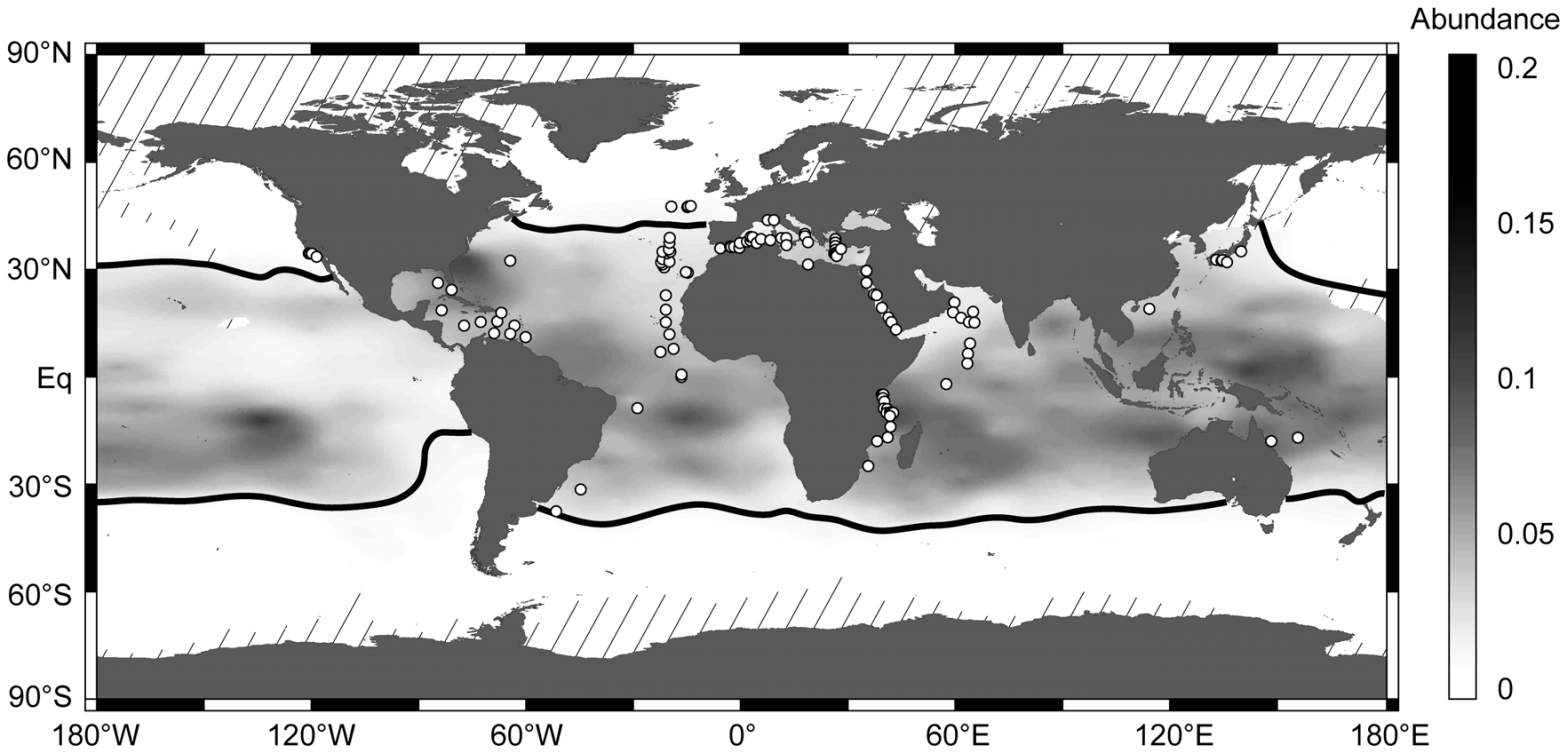
World map indicating the distribution of the target species and sampling sites for this study. Gray shading indicates the relative abundance of *Globigerinella siphonifera* as it is found in planktonic foraminiferal assemblages from surface sediments interpolated from data in the MARGO database [64] by Ocean Data View [65] in default projection. Black lines show the borders of occurrence with a threshold of 1%. White circles indicate the sampling stations of all samples included in this study. Diagonal lines indicate areas where no data are available.

In this study we present the results of a global survey on the foraminifera lineage *Globigerinella* [16], which is abundant in the surface waters in tropical and subtropical provinces throughout the world ocean (**Fig. 1**). The dominant morphospecies in this lineage, *G. siphonifera* tolerates a temperature range from 11°C to 30°C and a salinity range from 27–45‰ [17] and it is limited vertically to the euphotic zone due to its association with symbionts. In the modern ocean, *Globigerinella calida* [18] has been described as its sister species, but it is morphologically similar and its status as a separate species remains unclear. This study includes specimens that have been assigned to that species name as well. Within the typical *G. siphonifera* morphology, two divergent types were distinguished by different cellular morphology and symbionts [19], [20], and potentially also by morphological, physiological, chemical and genetic differences [21], [22]. The high degree of variability in the *G. siphonifera* lineage is reflected in its genetic diversity. Analyses of the small ribosomal subunit RNA gene (SSU rDNA), which is part of the only gene complex known so far in planktonic foraminifera, identified a large number of genetic lineages, which show no evidence for introgression and are typically considered as cryptic species [4], [21]–[24]. Based on these data, the *G. siphonifera* group appears to be the most genetically diverse lineage of modern planktonic foraminifera [4].

Although the existing sampling has been far from exhaustive, it seemed to indicate that individual cryptic genetic lineages within *G. siphonifera* are cosmopolitan [4], but their proportions vary with surface ocean properties [23]. Such distribution could be explained by a combination of unlimited dispersal and differential adaptation, but it remains uncertain whether it stands the test of global sampling. Here we analyze SSU rDNA sequence data from a global survey that covers the distribution range of *G. siphonifera* both latitudinally, across the tropical and subtropical oceans and their satellite semi-isolated marginal seas (**Fig. 1**) in order to study its biogeography and draw conclusions on the emergence of the observed high genetic diversity.

## Materials and Methods

### Ethics statement

The field collections carried out for the purpose of this paper did not involve endangered or protected species. Locations of all sampling stations are given in **Table S1**. The sampling was carried out in open ocean and followed the regulations for the exclusive economic zones (EEZ) of the coastal countries, provided for each expedition by the respective authority. No specific permission was required to collect the analyzed plankton.

### Sampling

Specimens of *Globigerinella siphonifera* were collected during 26 expeditions between 1996 and 2012 covering all seasons and water depths from the surface to 700 m (**Table S1**). The sampling represents a combination of plankton hauls during ship cruises, including stratified sampling, with nearshore collections by small nets and scuba diving. Mesh size varied from 100 to 200 μm. In all cases, individual foraminifera were separated from the rest of the plankton and taxonomically identified using stereomicroscopes. Living specimens still containing cytoplasm were cleaned using a brush and either transferred to 1.5 ml tubes for direct DNA extraction or air-dried on cardboard slides and stored at −20 or −80°C until further processing. In addition, the dataset was enhanced by inclusion of 45 sequences of *G. siphonifera* available in GenBank (**Table S1**). In order to resolve the phylogeny of the *G. siphonifera* sequences, to root the tree, and to estimate divergence times among the main lineages, we have attempted to obtain SSU rDNA sequences of the sister species *Beella digitata*. Eight specimens of that species have been collected from plankton nets in the Western Mediterranean (**Table S1**).

### DNA extraction, amplification and sequencing

DNA extraction followed either the DOC protocol of Holzmann & Pawlowski [25], during which the shell is dissolved, the guanidine method [26] or an urea method where the DNA is extracted in a mixture of 100 mM Tris (pH 8), 100 mM NaCl, 1% Sarcosyl, 8 M Urea and 2 mM TCEP, kept at room temperature. The latter two methods allow preservation of the shell. Polymerase chain reaction (PCR) was used to amplify a ∼350 to 1000 bp fragment of the 3′ end of the SSU rDNA either using the proofreading Vent® polymerase (New England Biolabs) or Taq DNA Polymerase (Qiagen). The amplified fragments include all sequence sites necessary to differentiate between the genetic lineages of *G. siphonifera*. Details on extraction, amplification and primers for all individuals are given in **Table S1**. PCR products were purified using the QIAquick gel extraction kit (Qiagen), Wizard® PCR clean up (Promega) or DNA Gel Extraction Kit (Millipore). Products were sequenced directly by external service providers (Agowa, Berlin and University of Edinburgh Gene Pool). In order to constrain intra-individual variability, eight individuals from different regions were cloned using the Zero Blunt® TOPO® PCR Cloning Kit (Invitrogen) with TOP10 chemically competent cells. Sequence chromatograms were checked manually for ambiguous reads and corrected where possible. All new sequences longer than 200 bp were submitted to GenBank (http://www.ncbi.nlm.nih.gov/; accession nos.: KF769560-KF769948).

### Delineation of genetic lineages

The primary sequence alignment was carried out in MAFFT v. 6.935b [27] using the option -auto, which allows the program to decide on the optimal alignment algorithm (**Alignment S2 in File S1**). Aurahs et al. [28] have shown that MAFFT handled best the particular sequence structure of foraminiferal SSU rDNA out of six alignment programs tested. The alignment was used to define the main genetic lineages and to group identical sequences (here referred to as ‘ribotypes’ (RT)), which present the same combination of certain sequence motifs within the amplified fragment of the SSU rDNA. This analysis identified the presence of three main lineages, which further split into up to seven clades. The initial automated alignment was split into three subalignments corresponding to the three main genetic lineages (**Alignments S4–6 in File S1**). For each subalignment sequence chromatograms were checked by eye for sequencing errors, sequence ends were trimmed and length-polymorphic regions were left-aligned by default in Mesquite v. 2.75 [29]. The SSU rDNA of foraminifera is characterized by the occurrence of highly length-polymorphic regions (LPR) in the core structure, which hinder the computation of straightforward alignments with consistent homology of individual base pairs [28]. The number of inferred parsimonious changes in these regions would be highly depending on the alignment, the hypothetical homology of individual sites. Therefore, we opted for treating each LPR as a single, complex character (an oligonucleotide motif) in the ribotype analysis instead.

Due to the different length of the individual accessions, and the particular nature of foraminifer expansions segments, the direct application of median-joining networks [30] to establish relationships between ribotypes of each major genetic lineage was not feasible. Instead ribotypes were analyzed based on the variable positions in each subalignment. Differing sequence patterns (point mutations and LPR motifs) were coded as a binary matrix, in which characters with more than two states were represented by a corresponding number of half-weighted binary characters. A point mutational pattern involving the nucleotides A, C, and G would be coded as 1 0 0, 0 1 0, and 0 0 1 using three characters with a weight of 5 instead of the standard weight of 10. LPR motifs were coded accordingly at this step. Mutation patterns that were only present in a single sequence were not considered separately, but merged with the nearest ribotype for abundance analysis. The resultant binary matrices comprising up to 19 ribotypes were then analyzed using NETWORK v. 4.5 (Fluxus Technologies Inc.) to compute median-joining (MJ) networks [30].

The recognition of ribotypes allowed us to structure the genetic diversity within *G. siphonifera* between the level of the three main lineages and the ribotypes into discrete and objectively defined genetic types, using a threshold of three mutational events. Ribotypes separated by three or fewer mutational events were considered to belong to the same genetic type. Earlier studies reported the existence of different ribotypes within the genome of one single individual in some but not all species of foraminifera [31]. Consistent with earlier investigations of intraindividual variability within the spinose planktonic clade [8], in our study, only one ribotype per individual was found, which was apparent by the lack of ambiguous sequence reads and was verified by cloning, which revealed identical sequences within single individuals. The apparent lack of hybridization among the ribotypes would suggest that they may represent genetically isolated units. However, we cannot entirely exclude the existence of hybrids with the present data because of insufficient cloning depth. Therefore, to avoid an over-interpretation of the genetic diversity and arrive at a number of distinguishable lineages, we reserve the (cryptic) species rank for genetic types.

### ML tree inference and bootstrapping

To resolve the phylogenetic relationships of the *G. siphonifera* lineages and *B. digitata* to the rest of the planktonic foraminifera, the MAFFT sequence alignment from Aurahs *et al.* [28], including sequences of 23 planktonic foraminifera morphospecies, was used as a basis to which the new sequences were aligned by the sequence adding function in MAFFT v. 7 [32] (**Alignment S1 in File S1**). Settings were left to default. This enlarged alignment was then used for tree inference under the maximum likelihood (ML) criterion with RAxML-HPC2 v. 7.6.3 [33] via the CIPRES Gateway [34]. The alignment was used without further manipulation or filtering. Branch support for the ML tree of the general foraminifera MAFFT alignment was established with the fast implementation (option –x) [35] of nonparametric bootstrapping (BS) [36]. The number of necessary replicates was determined by automatic bootstopping with the majority-rule tree based criterion (option -#autoMRE). The per-site rate approximation model [33] was used for the fast BS phase followed by a slow final model optimization under the general time reversible model allowing for between-site variation modeled via a gamma distribution (GTR + Г; option -m GTRCAT). Run parameters were set to infer in one run the best-known ML tree and perform a full BS analysis (option –f a).

To resolve further the relationships among the genetic types of *G. siphonifera*, a set of analyses has been carried out including only sequences of *G. siphonifera* and *B. digitata*. Following Aurahs *et al.* [28], the stability of the topology has been evaluated by a multiple alignment approach. To this end, automated alignments have been used, based on the default settings of the online-available, up-to-date versions of MAFFT v. 7, MUSCLE v. 3.7 [37] and KALIGN v. 2 [38]. Tree inference was conducted under the same settings as described above and without prior manual modification of the alignments.

### Molecular clock and speciation rates

In order to estimate the divergence time among the genetic lineages within *G. siphonifera*, a molecular clock approach was applied, using the *G. siphonifera*/*B. digitata* MAFFT alignment. *B. digitata* was used as an outgroup to define the *Globigerinella* root. Molecular clock analysis was performed using Bayesian methods implemented in BEAST v. 1.7.5. [39] via the CIPRES Gateway. The alignment was tested under various clock models (strict, uncorrelated lognormal and uncorrelated exponential). The split between *G. siphonifera* and *B. digitata* is marked in the fossil record by the first appearance of the species *Beella praedigitata* [40], [41]. This event is dated to 10.2 Ma in Aze *et al.* [41]; the age of the oldest reported occurrence of this species in deep-sea sediments is listed in the CHRONOS database as 11.96 Ma (http://chronos.org) [42]. Here we used the mean of the two ages (11.08 Ma) and associate this date with an uncertainty of 0.88 Ma. Detailed settings were the same for all three clock models tested. The distribution of the fixed node age prior was considered normal. The GTR+Г+I (adding a parameter for the proportion of invariant sites) was used as a substitution model, to allow for different evolutionary rates between variable and conserved regions of the SSU rDNA. Speciation rate was considered constant under the Yule-Process and a UPGMA tree was calculated as a starting tree. Markov-Chain-Monte Carlo (MCMC) analyses were conducted for 10,000,000 generations, with a burn-in of 1000 generations and saving each 1000^th^ generation. The maximum clade credibility tree with median node heights was calculated in TREEAnnotator from the BEAST package, with a burn-in of 100 trees and a posterior probability limit of 0.0. The resulting tree was then analyzed in FigTree v. 1.3.1 [43].

To test for trait dependency of changes in birth-only speciation rates among different clades, we applied a covariates generalized linear model (GLM) approach [44] on the trees produced by the lognormal and exponential uncorrelated clocks. This method allows to test, whether or not the presence of a certain trait had a significant effect on the speciation rate within given clades in a phylogenetic tree, taking branch-lengths into account. If reliable phylogenetic trees exist, it is considerably more powerful than traditional tests for changes in speciation rate, that only compare the number of lineages within adelphotaxa [45]. The test was performed in R v. 3.0.1 [46], using the package ‘ape’ v. 3.0.8 [47].

### Assessment of sampling intensity

For the global dataset and for the separate regions, first-order-Jackknifing [48], [49] was performed in R v. 3.0.1 to estimate the number of genetic types expected to occur in each region, given their occurrence in the sampling sites. Such test provides a first assessment whether or not the sampling was sufficient to detect all genetic lineages present in each region. For that, each station was treated as a separate sample, independent of the other stations, and it was assumed that the samples are sufficiently random and well distributed to allow such an approach, and cover the world ocean area to an extent that allows them to be assumed homogenous. The jackknifing is insofar most useful for this dataset, as it is fully independent of any possible interaction of different genetic types within the same quadrat, and offers a very good bias-correction for low densities per sample [49].

## Results

In addition to the 45 sequences from GenBank, in this study we obtained 370 partial sequences of the 3′ end of the SSU rDNA representing 338 individuals of *Globigerinella siphonifera* from 108 stations of 25 expeditions in seven regions of the world ocean (**Table S1**). The 3′ end of the SSU rDNA, routinely used in foraminifera molecular studies, includes the helices 32 to 49 [50] and additional foraminifera specific expansion segments of variable length. Most sequence divergence was found in the expansion segments 37/e1, 41/e1, 45/e1 and 46/e1, the variable region V7 consisting of several helices and the terminal part of helix 49 (Tp49) [51]. Furthermore, point mutations were also found in the sequentially and structurally conserved regions (helices 32–49) of foraminifera SSU rDNA (**Table S2**). All sequences obtained either by direct sequencing or cloning showed a clear signal and could be attributed without doubt to one of the main genetic lineages. We did not observe any intraindividual variability neither by seeing ambiguous reads at consistent positions or by observing variability among sequences from cloned specimens, which would be the case if individuals contained different ribotypes in the multiple copies of the SSU rDNA. Additionally, we obtained 25 sequences of eight individuals of *Beella digitata* covering the entire fragment of the SSU rDNA used for phylogenetic inference in planktonic foraminifera by Aurahs *et al.* [28].

**Figure 2:**
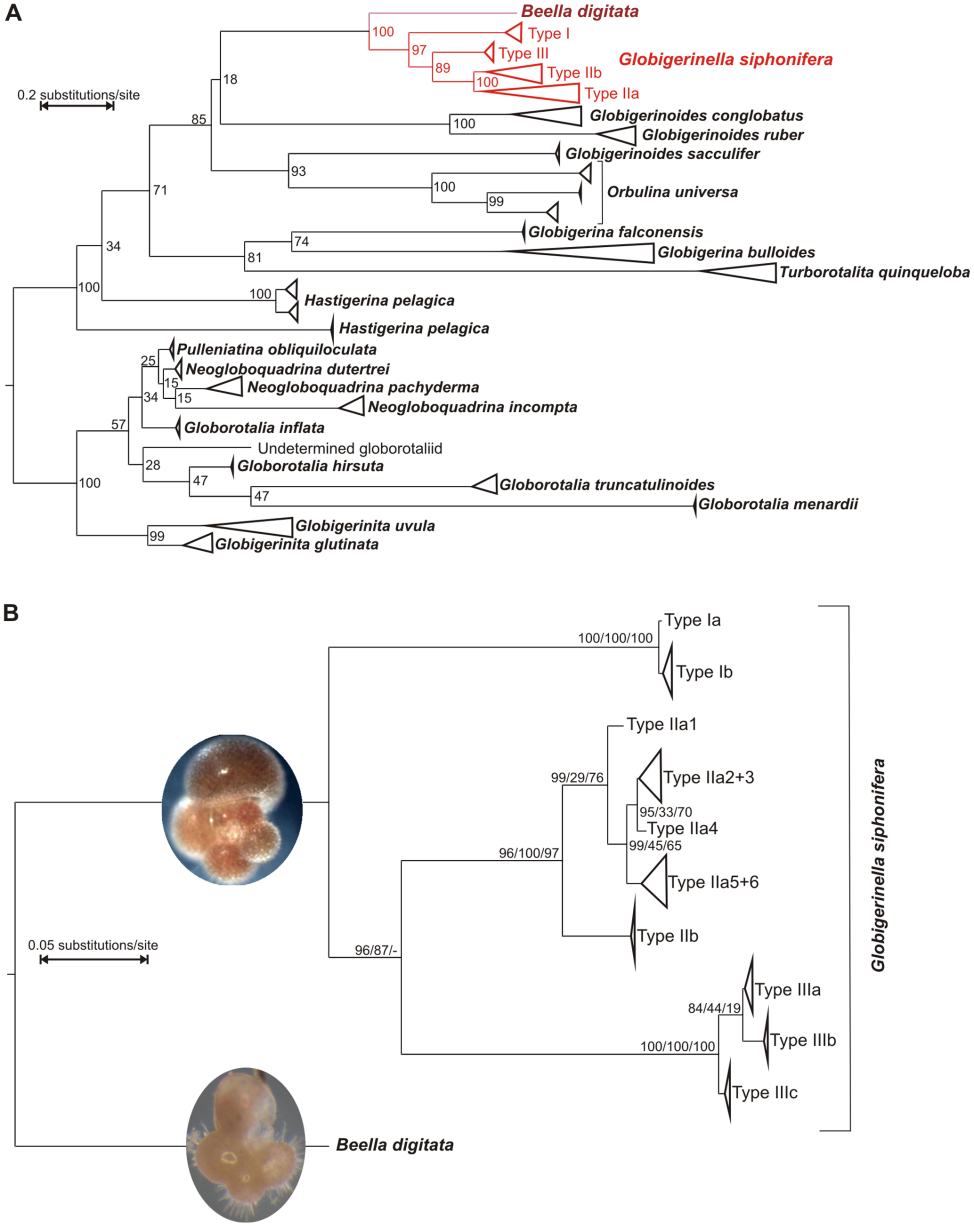
Phylogenetic relationships within planktonic foraminifera. **A**) Phylogenetic relationships of planktonic foraminifera including *Globigerinella siphonifera* and *Beella digitata*. The tree is based on the MAFFT alignment of Aurahs *et al.* [28] to which SSU rDNA sequences of *G. siphonifera* and *B. digitata* were added. Tree inference and calculation of bootstrap values was conducted in RAxML in the CIPRES gateway. Sequence diversity within morphospecies has been collapsed, except for *G. siphonifera* where only terminal branches were collapsed. **B**) Phylogenetic tree of *G. siphonifera* with *B. digitata* as an outgroup. The tree is based on a MAFFT alignment and was calculated in RAxML on the CIPRES gateway. Bootstrap values are shown based on MAFFT/MUSCLE/KALIGN alignments. Light microscopic images of *G. siphonifera* and *B. digitata* illustrate the gross morphology. Both individuals measure ∼250 μm across.

All sequences could be assigned to one of the three main lineages, which, applying a distance threshold of 0.1028, correspond to objectively definable taxonomic units [24]. These lineages are robust to increased taxonomic coverage, especially to the inclusion of *B. digitata* (**Fig. 2a**) and remain supported to >89% in maximum-likelihood inference. The sister relationship of *B. digitata* has been confirmed (**Fig. 2a**), supporting observations from the fossil record [40].

**Figure 3:**
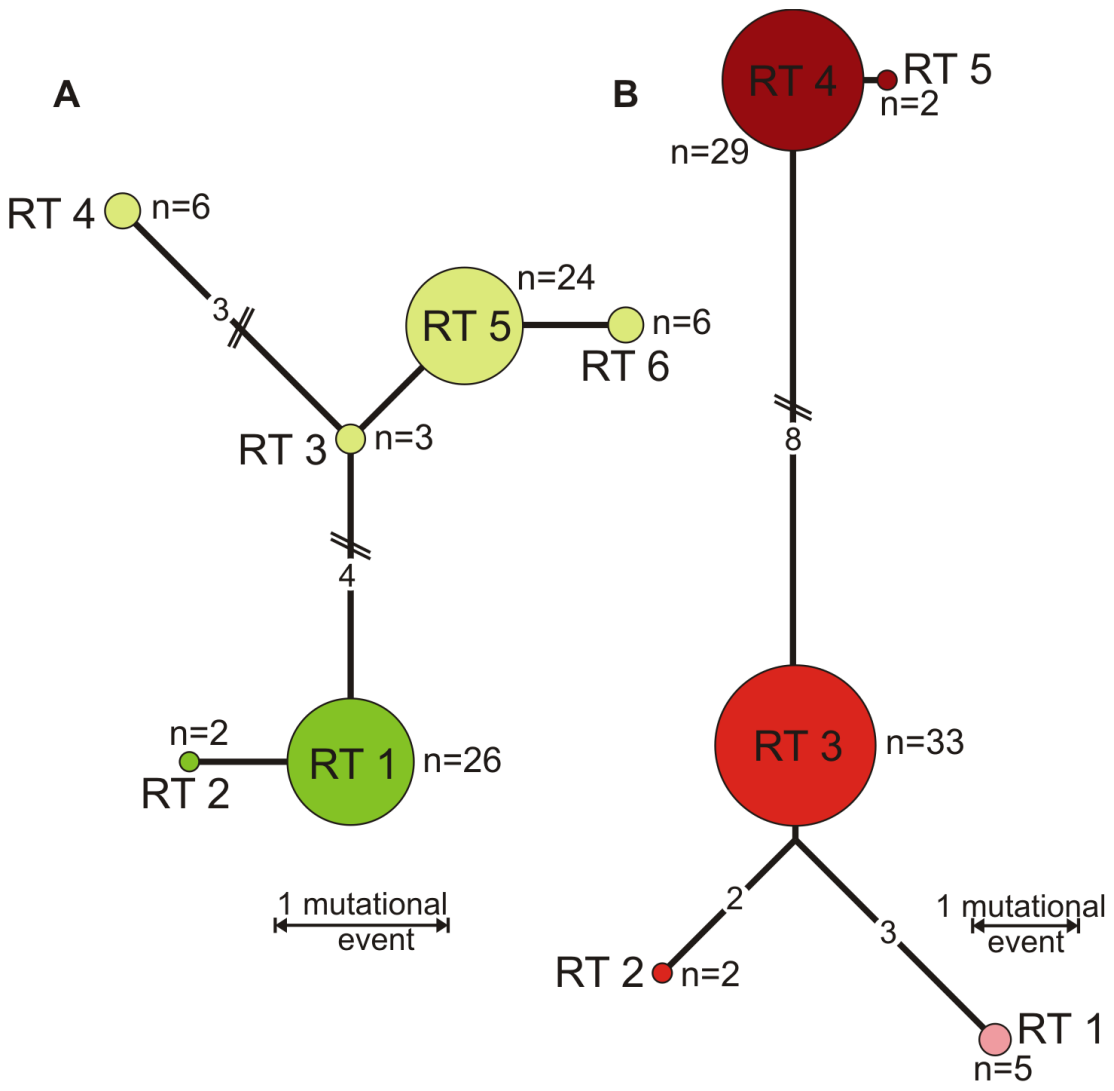
Ribotype networks for delineation of genetic types. **A**) Median-joining network of *Globigerinella siphonifera* lineage I showing genetic distances and relationships between ribotypes (RT) and their grouping into two basic genetic lineages, Ia (RT 1+2, bright green) and Ib (RT 3–6, light green). Numbers on links indicate amount of mutational events between two ribotypes if they are larger than one. n indicates number of individuals representing one ribotype. **B**) Ribotype network of lineage III distinguishing three basic lineages, IIIa (RT 1), IIIb (RT 2+3) and IIIc (RT 4+5), addressed by different shades of red.

**Figure 4:**
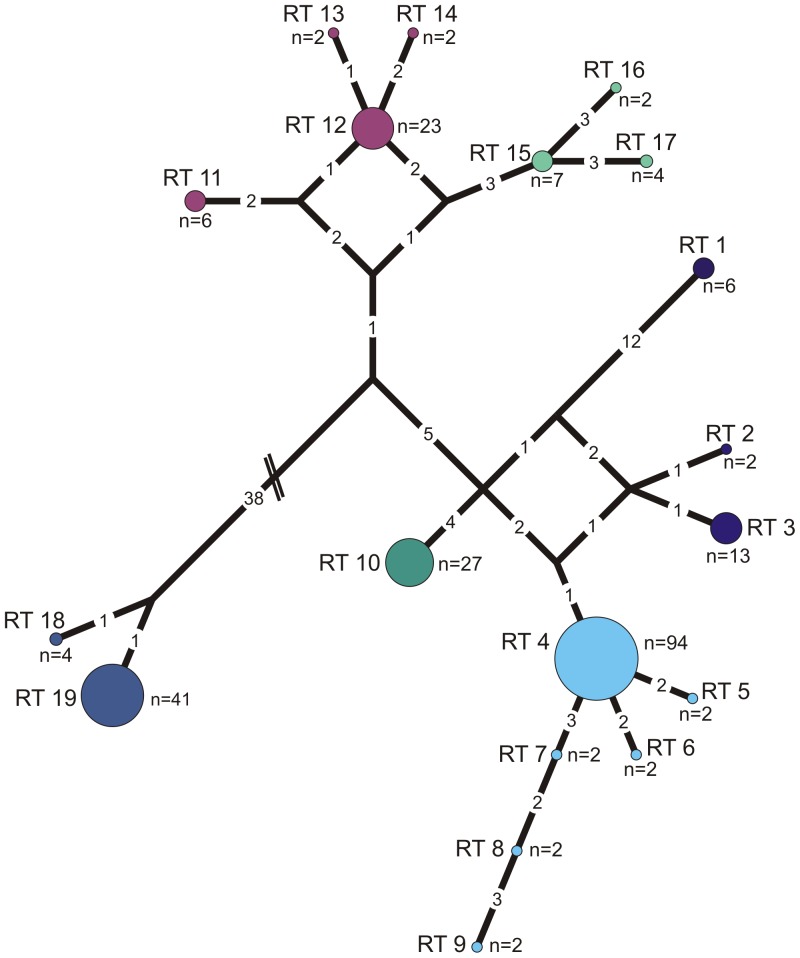
Ribotype network for delineation of genetic types. Ribotype network for *Globigerinella siphonifera* lineage II showing genetic distances and relationships between the 19 ribotypes (RT) and their assignment to different genetic lineages: IIa1 (RT 1), IIa2 (RT 2+3), IIa3 (RT 4–9), IIa4 (RT 10), IIa5 (RT 11–14), IIa6 (RT 15–17) and IIb (RT 18+19), addressed by different colors. Numbers at links indicate the number of mutational events between two ribotypes. n indicates number of individuals representing one ribotype.

Following the strict definition excluding singletons, the variability of the analyzed gene fragment of *G. siphonifera* reveals the existence of 30 SSU rDNA sequence variants (ribotypes; **Table S2**). This confirms the exceptional level of diversity noted in earlier studies [4]. Within lineage I, the six separated ribotypes can be organized into two basic genetic lineages, namely Ia (RT 1+2) and Ib (RT 3–6), that differ by up to eight characters (all of them point mutations; **Fig. 3a**). Mutations occur to equal parts in the variable regions (41/e1, 46/e1 and Tp49) and in the more conserved regions (helices 33, 36, 37, 43). The five ribotypes within lineage III are only little more divergent than those in lineage I, with two (RT 4+5) being separated by up to 13 point mutations from the remaining three (RT 1–3), which differ by 3–4 characters from each other (**Fig. 3b**). Consequently, these ribotypes can be classified into three different genetic lineages, IIIa, IIIb and IIIc. Mutations separating these lineages are exclusively point mutations and are mostly found in the variable regions (37/e1, 41/e1 and V7) and only in two conserved regions (helices 37 and 38). Highest divergence is found in lineage II, where sequence variation sums up to 19 ribotypes that can be grouped into seven genetic lineages (IIa1–6 and IIb; **Fig. 4**). RT 18 and 19 are with more than 40 mutational events most distinct and assigned to lineage IIb. Mutations in lineage II are homogeneously distributed between all variable and all conserved regions.

Subsequently, the phylogenetic relationships among the 30 ribotypes organized in 12 genetic lineages within *G. siphonifera* were tested using three different alignments (**Fig. 2b**). This analysis reveals that 10 out of the 12 genetic lineages, defined as differing by more than three characters, are supported in the majority of the alignments. A resolution down to the separate ribotypes as seen in the networks, however, is not possible in the tree, and therefore the terminal branches are collapsed. The topology of the phylogram, including the inferred allocation of mutation events to branches, indicates a nested, hierarchical pattern of divergence, suggesting an ongoing process of sequential differentiation.

It is remarkable that despite the seven-fold increase in sequencing effort compared to existing data, no new major lineages within *G. siphonifera* were discovered. A similar picture appears when individual genetic lineages are considered. Here, our data complement earlier studies [4], [23], [24] by discovering two new genetic lineages (lineages IIIb and c; **Fig. 2b**), which is again highly disproportionate to sequencing effort. At the lowest level of divergence considered, the proportion of newly discovered sequence motifs is the highest: 16 out of 30 ribotypes are reported here for the first time. Even here, the amount of ribotype discovery is disproportionate to sequencing effort and the higher number of new motifs simply reflects the hierarchical scaling within the clade.

**Figure 5:**
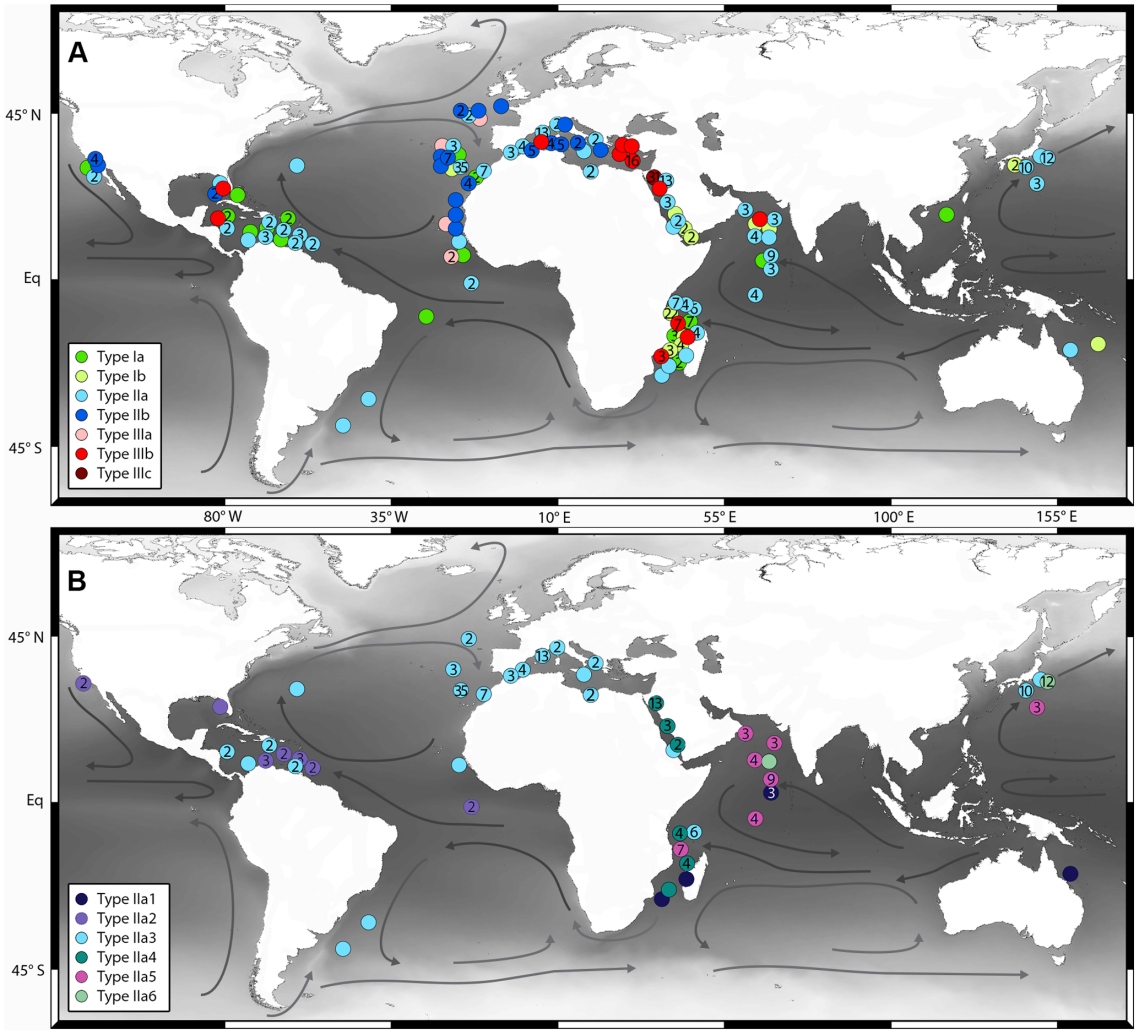
Biogeographic distribution of the genetic types of *Globigerinella siphonifera*. **A**) Geographic distribution of the *G. siphonifera* lineages plotted at their exact sampling locations on a map in Mercator projection. Numbers indicate the amount of individuals of one genetic type found at one station. One year mean sea surface temperature is indicated by gray shading. Arrows indicate main ocean currents. **B**) Geographic distribution of the genetic types of *G. siphonifera* lineage IIa.

The geographical distribution of specimens assigned to the twelve genetic lineages reveals the existence of cosmopolitanism as well as provincialism within cryptic genetic types of *G. siphonifera* (**Fig. 5a**). Type IIIc shows the most restricted occurrence; it was only found in the Gulf of Aquaba, where it has the highest abundance of all occurring types. Type IIIa was only found in low abundances and exclusively in the Eastern Atlantic. Type Ia seems to have a cosmopolitan occurrence since it was found in the majority of regions sampled. Types Ib, IIb and IIIb can also be considered cosmopolitan, although they are less evenly distributed. Type Ib has its highest abundances in the Western Indian Ocean and the Red Sea and very low abundances in the Atlantic, where only one individual was found. Type IIb was sampled in high numbers in the Atlantic, but only few individuals in the Eastern Pacific. Type IIIb was found in the marginal seas of the Atlantic and in the Western Indian Ocean.

The group of genetic types IIa is highly abundant globally and shows a truly cosmopolitan distribution. However, its constituent types show highly differentiated distribution patterns, characterized by a surprising difference in diversity between the Atlantic and the Pacific (**Fig. 5b**). The Indian Ocean contains the highest diversity with five different types of this lineage. Type IIa1 was found in very low abundances mainly in the Indian Ocean and one individual in the Coral Sea. Type IIa4 seems to be restricted to the Red Sea and the Western Indian Ocean. Type IIa5 is most abundant in the Arabian Sea, but also present in low numbers in the Northwestern Pacific. Type IIa6 was mainly found close to Japan, but apparently also occurs in the Indian Ocean as indicated by one individual sampled in the Arabian Sea. In contrast to the high diversity of lineage IIa in the Pacific and Indian Ocean, the diversity in the Atlantic is considerably more limited. There we only encountered two different types: Type IIa2, which except for two individuals off California seems to be restricted to the Atlantic Ocean and Type IIa3, which has a cosmopolitan distribution and occurs in every region sampled.

## Discussion

A surprisingly high SSU rDNA sequence divergence is found in most morphospecies of planktonic foraminifera [4]. This sequence divergence is typically organized into a small number of lineages, which show no evidence for hybridization, their divergences appear ancient and their distribution follows a geographical structure [10], [12]. For these reasons, such lineages, also referred to as “Types” or “Genetic types”, are considered to represent reproductively isolated taxonomic units akin to biological species. Although this interpretation appears most likely, it is fair to state that unambiguous evidence for the status of these lineages as biological species is lacking. This is because planktonic foraminifera do not reproduce in culture, so that cross-mating experiments such as those carried out for cryptic species of diatoms by Amato *et al.* [2] are at present impossible. Because of large differences in substitution rates, it is difficult to devise a universal threshold distance for DNA-based species delineation in the group [24]. However, evidence from existing surveys suggests that most divergences in the analyzed SSU rDNA fragment are not associated with hybridization. The lack of hybridization could be shown particularly well in cases where divergent multiple copies are found in sequences of SSU rDNA, or where additionally also the associated ITS region had been sequenced [12], [52]. On the other hand, an exhaustive survey of *Globigerinoides sacculifer*, a closely related species to *G. siphonifera*, revealed the existence of one rare divergent SSU sequence motif, which differed by three characters, but was associated with the same ITS sequence as specimens without the SSU motif [8]. Because of this observation and the divergence structure observed in our data (**Figs. 3**, **4**), we assume that the lowest level of genetic variability in *G. siphonifera*, manifested by the 30 SSU ribotypes, may not be associated with reproductive isolation, but represents divergence and rDNA variation within species. Because of the uncertainty in the interpretation of the evolutionary status of the 30 ribotypes, when analyzing the distribution of the 12 genetic lineages, which we consider cryptic species, we cannot be entirely sure that we are not underestimating the number of reproductively isolated lineages. However, since the difference in the distribution and allocation of cryptic diversity is manifested already at the level of the 12 genetic lineages, the conclusions drawn from the lineage-level data must also apply to any unit below these.

Notwithstanding the exact status of the 12 genetic lineages, the first step before analyzing their distribution and allocation is to ask how representative the sampling has been. To this end, the first-order-Jackknifing approach (**Table 1**), which serves as an objective estimate of lineage richness that is to be expected both globally and regionally, shows that the number of lineages in our collection appears to approach the expected total number of lineages, given the assumptions of the test. Similarly, the number of sampled lineages in almost every region falls within the 95% confidence interval of the Jackknifing estimate, implying that further lineages are unlikely to have been discovered in each region by more intensive sampling. Only for the Red Sea does the test indicate the existence of at least one lineage that has not been sampled yet. This analysis confirms the empirical observation that a seven-fold increase in sampling intensity led to a disproportionately low rate of discovery of new variants and that the distribution of the proportion of new variants is scaled with their hierarchical position. Despite the higher lineage diversity than among other planktonic foraminifera species (12 in *G. siphonifera*, compared to 7 in *Neogloboquadrina pachyderma* and *Globigerina bulloides* [4]), the global survey in the “hyperdiverse” *G. siphonifera* confirms, that the total number of cryptic genetic types within morphospecies of planktonic foraminifera is limited and that the biological diversity in the group may be underestimated by a factor of about 10, but not significantly more.

**Table 1:** Comparison between observed and estimated number of genetic types.

Region	*S_o_*	*S_e_*	*CI_95_*	*S_o_∈ S_e_ ± CI_95_*
Global	12	12.99	1.95	true
Atlantic Ocean	6	6.97	1.90	true
Mediterranean Sea	3	3	0	true
Caribbean Sea	5	5.93	1.83	true
Red Sea	5	7.67	2.61	false
Arabian Sea	6	8.73	2.76	true
Western Indian Ocean	7	7	0	true
Pacific Ocean	8	8.96	1.89	true

Observed (*S_o_*) and estimated (*S_e_*, first-order-Jackknifing) number of genetic types of *Globigerinella siphonifera* for the global and regional data sets. Only in the Red Sea the observed number of types does not fall within the 95% confidence interval (CI_95_) of the estimate, suggesting the existence of at least one more genetic type in that region.

Observed (*S_o_*) and estimated (*S_e_*, first-order-Jackknifing) number of genetic types of *Globigerinella siphonifera* for the global and regional data sets. Only in the Red Sea the observed number of types does not fall within the 95% confidence interval (CI_95_) of the estimate, suggesting the existence of at least one more genetic type in that region.

Having established that the sampling intensity, both globally and regionally, can reasonably be considered sufficient to capture the occurrence pattern of the *G. siphonifera* lineages, we first consider the relationships of these lineages within the phylogenetic tree. Here, a major finding is the uneven distribution of diversification between the three main lineages; with seven types in lineage II and only two and three types in lineage I and III respectively. Since the Jackknifing analysis suggests that our sampling approaches the real diversity in each region, the uneven distribution of types between the lineages is unlikely to be due to systematic undersampling.

**Figure 6:**
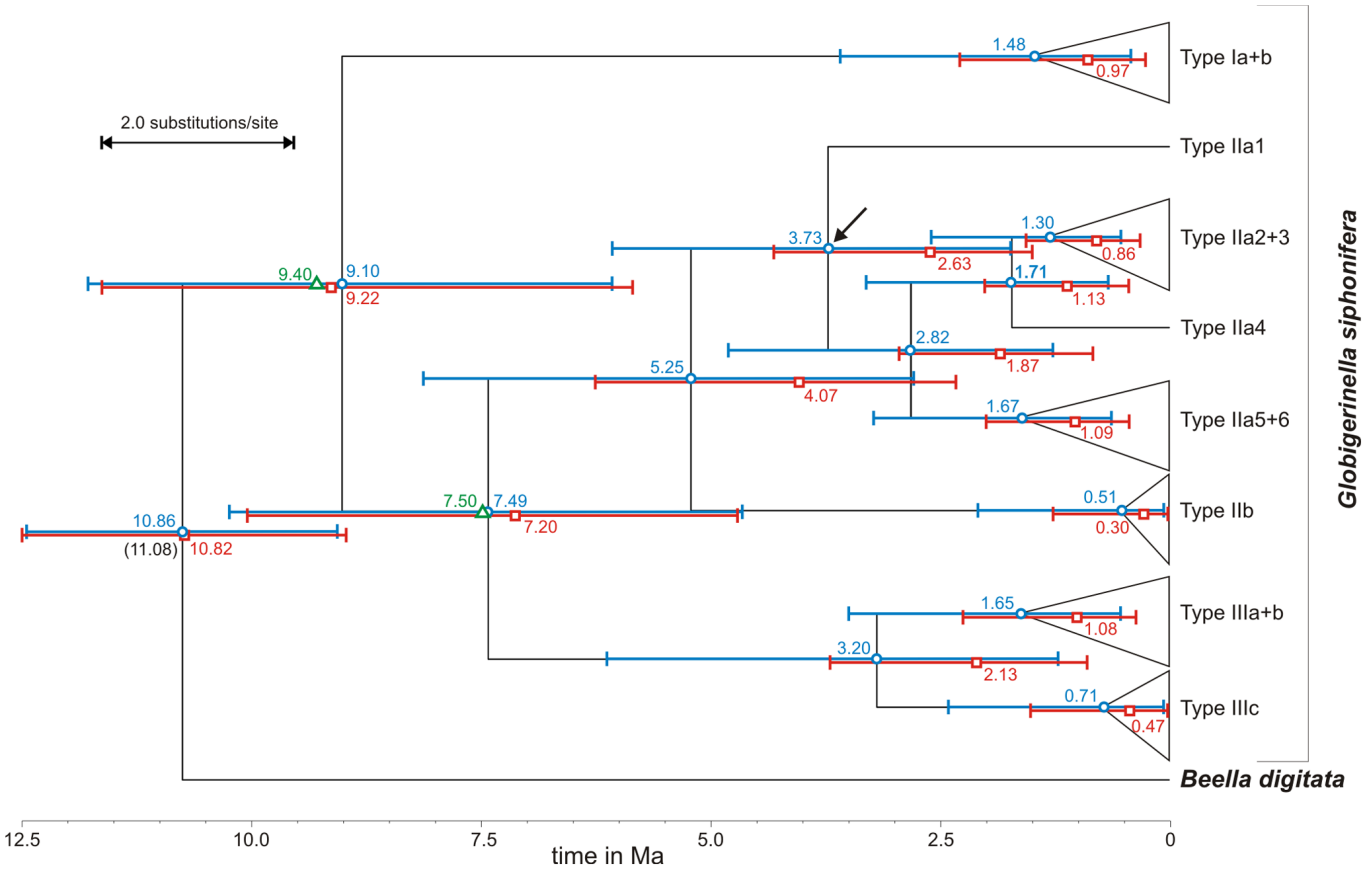
Molecular clock estimates for the evolution of the *Globigerinella siphonifera* lineages. Molecular phylogeny of *G. siphonifera* and *Beella digitata* based on a MAFFT alignment with time estimate ranges from the uncorrelated lognormal (blue) and exponential (red) molecular clocks. Numbers at nodes indicate the divergence ages shown with their 95% confidence intervals. Number in brackets indicates fixed age for the split of *G. siphonifera* and *B. digitata*. Green triangles and numbers show ages calculated in de Vargas *et al.* [23], except for one terminal node which seems too young. Black arrow indicates the starting point from where the presence of a certain trait had a significant effect on the speciation rate, based on a covariates generalized linear model approach.

The second obvious explanation for uneven allocation of diversity to lineages is their age, with older lineages having more time to accumulate species [53]. To test this hypothesis, we calculated molecular clocks for the diversification of genetic lineages within *G. siphonifera* based on the dating of the split from its sister species *B. digitata* (**Fig. 6**) [40], [41]. The ages resulting from both relaxed clock models showed a more realistic distribution than the results of a strict clock model and agree remarkably well with earlier calculations based on entirely independent calibrations [23]. The age for the split of the hyperdiverse lineage IIa from lineage IIb is calculated to have taken place ∼5 Ma in the early Pliocene. The split between lineage II and III dates to ∼7 Ma and the split of lineage I from the rest of the lineages took place ∼9 Ma. Thus, as the branching order of the phylogeny alone indicates (**Fig. 2**), the highest number of genetic types is found in the youngest lineage. Based on the molecular clock estimates (**Fig. 6**), this lineage had a two to three times shorter duration than the other lineages. In consequence, lineage longevity is not feasible as an explanation for unbalanced distribution of diversity.

Thus, since the high diversity in lineage II is unlikely to be a result of undersampling and is not correlated with lineage age, we may consider the possibility of it resulting from uneven rates of diversification among the lineages [54]. We test this hypothesis by using a covariates GLM approach that analyzes trait dependency of changes in birth-only speciation rates. The results reveal that speciation rates in lineage IIa must have been significantly higher than in all other lineages within *G. siphonifera*. This result is consistent for the uncorrelated lognormal (*χ* ^2^ = 4.258, df = 1, *p* = .039) as well as the exponential (*χ* ^2^ = 8.232, df = 1, *p* = .004) molecular clock analysis. Thus, we conclude that increased speciation rate seems most likely to be the cause for the disproportionate accumulation of diversity that occurred in lineage IIa.

The exact factor causing an increase in speciation rate in the hyperdiverse lineage IIa is difficult to reconstruct from the phylogeny alone. However the topology of the median joining network of lineage II (**Fig. 4**) reveals a centripetal distribution of ribotypes, with missing ancestral motifs. Such distribution implies that lineage II diversified by sequential fragmentation of a population of ancestral ribotypes, which was entirely transformed during the fragmentation process. This is interesting because it speaks against speciation by peripheral isolation.

The second clue to the unique status of the hyperdiverse lineage IIa comes from its biogeography. The striking pattern of (Indo-)Pacific isolation within this lineage (Types IIa1, 4–6; **Fig. 5**) has not only consequences for the interpretation of its elevated diversity, but it offers critical evidence to evaluate the biogeography of the cryptic genetic diversity of the constituent morphological species. To this end, we consider the three end-member scenarios explaining restricted distribution in turn (dispersal limitation, differential adaptation or niche incumbency).

First, we argue that the biogeographic distribution of the genetic lineages of *G. siphonifera* (**Fig. 5**) shows that a dispersal limitation does not seem to be the likely factor causing divergence in this taxon. In every one of the three lineages we find at least one type with a cosmopolitan distribution. Even the hyperdiverse lineage IIa contains one type (IIa3) with a global occurrence. If dispersal limitation would be the prevailing factor for speciation, we should expect an accumulation of endemic types in the Atlantic. The connection between the tropical-subtropical Atlantic and Indopacific habitats of *G. siphonifera* (**Fig. 1**) is mediated by the Agulhas current, which transports warm saline water from the Indopacific to the Atlantic [55] and was shown to carry live populations of planktonic foraminifera with it [56]. Therefore, in theory, lineages originating in the Atlantic should be much less likely to be able to escape from there, whereas lineages originating in the Indopacific should be constantly passively transported to the Atlantic due to the absence of a dispersal barrier. Indeed, for some species of marine copepods genetic differentiation and isolation of Atlantic populations due to limited dispersal between ocean basins were shown [57], whereas other species revealed a cosmopolitan distribution with a lack of barriers to gene flow and also showed a connection between the Indian Ocean and the Southern Atlantic [58]. These studies revealed no evidence for a population isolated in the Pacific Ocean and the observed biogeography thus could be considered consistent with passive dispersal.

The similarity of relative abundances of genetic lineages in *Globigerinella* between the different ocean basins analyzed by non-metric multidimensional scaling (**Figure S1**) reveals a close relationship between the Atlantic Ocean with its marginal seas, the Mediterranean and the Caribbean Sea. Also the Arabian Sea and its neighboring region, the Western Indian Ocean, show a high similarity in genetic type occurrence as well as the Red Sea which is affected by inflowing water from the Arabian Sea. The analysis shows the Pacific community to be related similarly to the Atlantic as well as to the Indian Ocean, however there is no close similarity between the Indian Ocean and the Atlantic. This observation is completely contrary to what would be expected if the occurrence of genetic lineages reflected passive dispersal by currents between the Atlantic and the Indian Ocean. Our conclusion that dispersal limitation is unlikely the cause of the observed pattern is in line with widespread evidence for global mixing in tropical populations of other species of planktonic foraminifera [8], [11] as well as evidence based on observations in the fossil record [59].

Second, we consider ubiquitous dispersal and differential adaptation. The accumulation of genetic types in the Indopacific could be indicative for differential adaptation of these genetic types to ecological or hydrographical conditions which are only realized in this region. We consider this explanation unlikely, because all of the endemic genetic types co-occurred upon collection in the same samples with genetic types that are cosmopolitan and there was no systematic offset in living depth among any of the genetic types, as evidenced by their occurrence in stratified plankton hauls. Further, types IIa2 and IIa3, which show a wider distribution or even are cosmopolitan, are nested within the clade comprising the endemic types. If there was a specific adaptation associated within the hyperdiverse lineage that limits its occurrence to the Indopacific then two independent evolutionary events are required to have occurred: the character had to evolve at the base of the IIa clade and then be reversed at the base of the IIa2 + IIa3 clade.

Therefore the most likely scenario to explain the distribution of the genetic types in the hyperdiverse lineage is the concept of niche incumbency [5], [60]. In this scenario, we assume that the diversification of lineage IIa has taken place in the (Indo-)Pacific by sequential fragmentation of the parent population. Until the divergence of the IIa2 + IIa3 clade, all lineages either remained restricted to the Indopacific or their invasion efforts into the Atlantic ended in extinction. The reason for the failure of most of the genetic types in this lineage to spread into the Atlantic would be incumbency – the niche that these genetic types possess is strongly overlapping with that of an Atlantic incumbent (whichever it may be), preventing the Pacific invaders, carried with the Agulhas current, to establish a viable population in the Atlantic. On a smaller scale, an exclusion pattern may in fact be expressed in the Atlantic between the invasive types IIa2 and IIa3 which represent two closely related sister lineages. The majority of individuals of Type IIa3 were found in the Eastern Atlantic and the Mediterranean Sea, whereas type IIa2 is the dominant type in the western part of the North Atlantic and the Caribbean. Requiring only one evolutionary event (the ability of the IIa2 + IIa3 lineage to invade the Atlantic), the niche incumbency or competitive exclusion thus seems to be a more parsimonious explanation of the distribution pattern of the genetic lineages of *G. siphonifera*.

The unexpectedly high genetic diversity as well as the differentiated distribution of the genetic types in the studied planktonic foraminifera show that occurrence patterns based on morphological species are too coarse to elucidate biogeographic patterns. In agreement with previous studies [11], [12], we show that the differentiated pattern of lineage distribution is unlikely to reflect dispersal limitation, but that it also does not simply reflect passive dispersal by ocean currents. Instead, these results confirm that even in marine microplankton high diversification is possible [61] and that interactions and competition between lineages together with historical contingency shape their present-day occurrence and distribution in the world ocean.

## Supporting Information

Figure S1 Rendition of similarity of relative abundances of all genetic types of *G. siphonifera* in the sampling regions. In order to statistically assess the geographical structure in the occurrence of genetic lineages of *G. siphonifera*, the sampling sites were separated into seven regions of the world ocean. The similarity of relative abundances of genetic lineages among these regions was visualized using non-metric multidimensional scaling based on the Morisita similarity index [62], as implemented in the PAST software v. 2. 17c [63]. Arrows indicate the direction of surface ocean currents connecting neighboring regions.
(TIF)

Table S1 Information on individual samples and handling procedures. Detailed information on each *G. siphonifera* individual used in the study (Sheet 1), GenBank samples added to the dataset (Sheet 2) and primer table with all different primers used (Sheet 3).
(XLSX)

Table S2 Sequence differences between *G. siphonifera* ribotypes. Table showing the sequence differences and their location in the secondary structure of the SSU rDNA used for differentiation of ribotypes within the three main lineages.
(XLSX)

File S1 Sequence alignments used for phylogenetic reconstructions and delineation of genetic types. MAFFT alignment of sequences of 23 planktonic foraminifera morphospecies including representative sequences of every ribotype of *G. siphonifera* and *B. digitata* from this study (Alignment S1); MAFFT alignment of all *G. siphonifera* sequences used in this study including GenBank sequences (Alignment S2); MAFFT alignment of representative sequences of every ribotype of *G. siphonifera* and *B. digitata* (Alignment S3) and *G. siphonifera* subalignments for each of the three major lineages (Alignment S4-S6).
(ZIP)

## References

[1] Šlapeta J, López-García P, Moreira D (2006) Global dispersal and ancient cryptic species in the smallest marine eukaryotes. Mol Biol Evol 23: 23–29. 16120798 10.1093/molbev/msj001

[2] Amato A, Kooistra WHCF, Levialdi Ghiron JH, Mann DG, Pröschold T, et al (2007) Reproductive isolation among sympatric cryptic species in marine diatoms. Protist 158: 193–207. 17145201 10.1016/j.protis.2006.10.001

[3] Bickford D, Lohman DJ, Sodhi NS, Ng PKL, Meier R, et al (2007) Cryptic species as a window on diversity and conservation. Trends Ecol Evol 22: 148–155. 17129636 10.1016/j.tree.2006.11.004

[4] Darling KF, Wade CM (2008) The genetic diversity of planktic foraminifera and the global distribution of ribosomal RNA genotypes. Mar Micropaleontol 67: 216–238.

[5] Algar AC, Mahler DL, Glor RE, Losos JB (2013) Niche incumbency, dispersal limitation and climate shape geographical distributions in a species-rich island adaptive radiation. Global Ecol Biogeogr 22: 391–402.

[6] Palumbi SR (1994) Genetic divergence, reproductive isolation, and marine speciation. Ann Rev Ecol Syst 25: 547–572.

[7] Hemleben C, Spindler M, Anderson OR (1989) Modern planktonic foraminifera. Heidelberg: Springer.

[8] André A, Weiner A, Quillévéré F, Aurahs R, Morard R, et al (2013) The cryptic and the apparent reversed: Lack of genetic differentiation within the morphologically diverse plexus of the planktonic foraminifer *Globigerinoides sacculifer* . Paleobiol 39: 21–39.

[9] Darling KF, Wade CM, Stewart IA, Kroon D, Dingle R, et al (2000) Molecular evidence for genetic mixing of Arctic and Antarctic subpolar populations of planktonic foraminifers. Nature 405: 43–47. 10811211 10.1038/35011002

[10] Aurahs R, Grimm GW, Hemleben V, Hemleben C, Kucera M (2009) Geographical distribution of cryptic genetic types in the planktonic foraminifer *Globigerinoides ruber* . Mol Ecol 18: 1692–1706. 19302352 10.1111/j.1365-294X.2009.04136.x

[11] Weiner A, Aurahs R, Kurasawa A, Kitazato H, Kucera M (2012) Vertical niche partitioning between cryptic sibling species of a cosmopolitan marine planktonic protist. Mol Ecol 21: 4063–4073. 22738662 10.1111/j.1365-294X.2012.05686.x

[12] Morard R, Quillévéré F, Douady CJ, de Vargas C, de Garidel-Thoron T, et al (2011) Worldwide genotyping in the planktonic foraminifer *Globoconella inflata*: Implications for life history and paleoceanography. PLOS ONE 6: e26665. 22028935 10.1371/journal.pone.0026665 PMC3197684

[13] de Vargas C, Norris R, Zaninetti L, Gibb SW, Pawlowski J (1999) Molecular evidence of cryptic speciation in planktonic foraminifers and their relation to oceanic provinces. Proc Natl Acad Sci USA 96: 2864–2868. 10077602 10.1073/pnas.96.6.2864 PMC15860

[14] Darling K, Kucera M, Wade C (2007) Global molecular phylogeography reveals persistent Arctic circumpolar isolation in a marine planktonic protist. Proc Natl Acad Sci USA 104: 5002–5007. 17360336 10.1073/pnas.0700520104 PMC1829254

[15] Seears H, Darling K, Wade C (2012) Ecological partitioning and diversity in tropical planktonic foraminifera. BMC Evol Biol 12: 54. 22507289 10.1186/1471-2148-12-54 PMC3361484

[16] d’Orbigny A (1839) Foraminifèrs. In: de la Sagra R, editor. Histoire Physique et Naturelle de L’ile de Cuba. Paris: Bertrand A. pp. 82.

[17] Bijma J, Faber WW, Hemleben C (1990) Temperature and salinity limits for growth and survival of some planktonic foraminifers in laboratory cultures. J Foramin Res 20: 95–116.

[18] Parker FL (1962) Planktonic foraminiferal species in pacific sediments. Micropaleontol 8: 219–254.

[19] Faber WW, Anderson OR, Lindsey JL, Caron DA (1988) Algal-foraminiferal symbiosis in the planktonic foraminifer *Globigerinella aequilateralia*; I, Occurrence and stability of two mutually exclusive chrysophyte endosymbionts and their ultrastructure. J Foramin Res 18: 334–343.

[20] Faber WW, Anderson OR, Caron DA (1989) Algal-foraminiferal symbiosis in the planktonic foraminifer *Globigerinella aequilateralis*; II, Effects of two symbiont species on foraminiferal growth and longevity. J Foramin Res 19: 185–193.

[21] Huber BT, Bijma J, Darling KF (1997) Cryptic speciation in the living planktonic foraminifer *Globigerinella siphonifera* (d' Orbigny). Paleobiol 23: 33–62.

[22] Bijma J, Hemleben C, Huber BT, Erlenkeuser H, Kroon D (1998) Experimental determination of the ontogenetic stable isotope variability in two morphotypes of *Globigerinella siphonifera* (d'Orbigny). Mar Micropaleontol 35: 141–160.

[23] de Vargas C, Bonzon M, Rees NW, Pawlowski J, Zaninetti L (2002) A molecular approach to biodiversity and biogeography in the planktonic foraminifer *Globigerinella siphonifera* (d'Orbigny). Mar Micropaleontol 45: 101–116.

[24] Göker M, Grimm GW, Auch AF, Aurahs R, Kucera M (2010) A clustering optimization strategy for molecular taxonomy applied to planktonic foraminifera SSU rDNA. Evol Bioinform 6: 97–112. 10.4137/ebo.s5504 PMC2964048 21037964

[25] Holzmann M, Pawlowski J (1996) Preservation of foraminifera for DNA extraction and PCR amplification. J Foramin Res 26: 264–267.

[26] Morard R, Quillévéré F, Escarguel G, Ujiie Y, de Garidel-Thoron T, et al (2009) Morphological recognition of cryptic species in the planktonic foraminifer *Orbulina universa* . Mar Micropaleontol 71: 148–165.

[27] Katoh K, Kuma K-i, Toh H, Miyata T (2005) MAFFT version 5: improvement in accuracy of multiple sequence alignment. Nucleic Acids Res 33: 511–518. 15661851 10.1093/nar/gki198 PMC548345

[28] Aurahs R, Göker M, Grimm GW, Hemleben V, Hemleben C, et al (2009) Using the multiple analysis approach to reconstruct phylogenetic relationships among planktonic foraminifera from highly divergent and lenght-polymorphic SSU rDNA sequences. Bioinform Biol Insights 3: 155–177. 20140067 10.4137/bbi.s3334 PMC2808177

[29] Maddison WP, Maddison DR (2011) Mesquite: a modular system for evolutionary analysis. Version 2.75. Available: http://mesquiteproject.org/mesquite/mesquite.html

[30] Bandelt HJ, Forster P, Röhl A (1999) Median-joining networks for inferring intraspecific phylogenies. Mol Biol Evol 16: 37–48. 10331250 10.1093/oxfordjournals.molbev.a026036

[31] Pillet L, Fontaine D, Pawlowski J (2012) Intra-Genomic Ribosomal RNA Polymorphism and morphological variation in *Elphidium macellum* suggests inter-specific hybridization in Foraminifera. PLOS ONE 7: e32373. 22393402 10.1371/journal.pone.0032373 PMC3290570

[32] Katoh K, Standley DM (2013) MAFFT Multiple Sequence Alignment Software Version 7: Improvements in performance and usability. Mol Biol Evol 30: 772–780. 23329690 10.1093/molbev/mst010 PMC3603318

[33] Stamatakis A. Phylogenetic models of rate heterogeneity: a high performance computing perspective. Proceedings of the IPDPS; 2006; Rhodos, Greece.

[34] Miller MA, Pfeiffer W, Schwartz T (2010) Creating the CIPRES Science Gateway for inference of large phylogenetic trees. Proceedings of the Gateway Computing Environments Workshop (GCE): 1–8.

[35] Stamatakis A, Hoover P, Rougemont J (2008) A rapid bootstrap algorithm for the RAxML web servers. Syst Biol 57: 758–771. 18853362 10.1080/10635150802429642

[36] Felsenstein J (1985) Confidence limits on phylogenies: An approach using the Bootstrap. Evolution 39: 783–791. 28561359 10.1111/j.1558-5646.1985.tb00420.x

[37] Edgar RC (2004) MUSCLE: multiple sequence alignment with high accuracy and high throughput. Nucleic Acids Res 32: 1792–1797. 15034147 10.1093/nar/gkh340 PMC390337

[38] Lassmann T, Frings O, Sonnhammer ELL (2009) Kalign2: high-performance multiple alignment of protein and nucleotide sequences allowing external features. Nucleic Acids Res 37: 858–865. 19103665 10.1093/nar/gkn1006 PMC2647288

[39] Drummond A, Rambaut A (2007) BEAST: Bayesian evolutionary analysis by sampling trees. BMC Evol Biol 7: 214. 17996036 10.1186/1471-2148-7-214 PMC2247476

[40] Kennett JP, Srinivasan MS (1983) Neogene planktonic Foraminifera: A phylogenetic atlas. Stroudsburg, PA: Hutchinson Ross Publishing Co.

[41] Aze T, Ezard THG, Purvis A, Coxall HK, Stewart DRM, et al (2011) A phylogeny of Cenozoic macroperforate planktonic foraminifera from fossil data. Biol Rev Camb Philos Soc 86: 900–927. 21492379 10.1111/j.1469-185X.2011.00178.x

[42] Kucera M, Schönfeld J (2007) The origin of modern oceanic foraminiferal faunas and Neogene climate change. In: Williams M, Haywood AM, Gregory FJ, Schmidt DN, editors. Deep-Time Perspectives on Climate Change: Marrying the Signal from Computer Models and Biological Proxies. London: The Geological Society. pp. 409–426.

[43] Rambaut A (2009) Tree Figure Drawing Tool Version 1.3.1. University of Edinburgh. Available: http://tree.bio.ed.ac.uk/software/figtree/.

[44] Paradis E (2005) Statistical analysis of diversification with species traits. Evolution 59: 1–12. 15792222

[45] Paradis E (2011) Shift in diversification in sister-clade comparisons: A more powerful test. Evolution 66: 288–295. 22220883 10.1111/j.1558-5646.2011.01429.x

[46] R Development Core Team (2011) R: A language and environment for statistical computing. Vienna: R Foundation for Statistical Computing.

[47] Paradis E, Claude J, Strimmer K (2004) APE: Analyses of Phylogenetics and Evolution in R language. Bioinformatics 20: 289–290. 14734327 10.1093/bioinformatics/btg412

[48] Quenouille MH (1949) Approximate tests of correlation in time-series. J R Stat Soc Series B Stat Methodol 11: 68–84.

[49] Smith EP, van Belle G (1984) Nonparametric estimation of species richness. Biometrics 40: 119–129.

[50] Wuyts J, Van de Peer Y, Winkelmans T, De Wachter R (2002) The European database on small subunit ribosomal RNA. Nucleic Acids Res 30: 183–185. 11752288 10.1093/nar/30.1.183 PMC99113

[51] Grimm GW, Stögerer K, Topaç Ertan K, Kitazato H, Kučera M, et al (2007) Diversity of rDNA in Chilostomella: Molecular differentiation patterns and putative hermit types. Mar Micropaleontol 62: 75–90.

[52] Quillévéré F, Morard R, Escarguel G, Douady CJ, Ujiié Y, et al. (2013) Global scale same-specimen morpho-genetic analysis of *Truncorotalia truncatulinoides*: A perspective on the morphological species concept in planktonic foraminifera. Palaeogeogr Palaeocl: http://dx.doi.org/10.1016/j.palaeo.2011.1003.1013, (in press, Corrected Proof).

[53] McPeek MA, Brown JM (2007) Clade age and not diversification rate explains species richness among animal taxa. Am Nat 169: E97–E106. 17427118 10.1086/512135

[54] Rabosky DL, Donnellan SC, Talaba AL, Lovette IJ (2007) Exceptional among-lineage variation in diversification rates during the radiation of Australia's most diverse vertebrate clade. Proc R Soc B 274: 2915–2923. 10.1098/rspb.2007.0924 PMC2291154 17878143

[55] Beal LM, De Ruijter WPM, Biastoch A, Zahn R (2011) On the role of the Agulhas system in ocean circulation and climate. Nature 472: 429–436. 21525925 10.1038/nature09983

[56] Peeters FJC, Acheson R, Brummer GJA, de Ruijter WPM, Schneider RR, et al (2004) Vigorous exchange between the Indian and Atlantic oceans at the end of the past five glacial periods. Nature 430: 661–665. 15295596 10.1038/nature02785

[57] Goetze E (2011) Population Differentiation in the Open Sea: Insights from the Pelagic Copepod *Pleuromamma xiphias* . Integrative and Comparative Biology 51: 580–597. 21940778 10.1093/icb/icr104

[58] Blanco-Bercial L, Álvarez-Marqués F, Bucklin A (2011) Comparative phylogeography and connectivity of sibling species of the marine copepod Clausocalanus (Calanoida). Journal of Experimental Marine Biology and Ecology 404: 108–115.

[59] Sexton PF, Norris RD (2008) Dispersal and biogeography of marine plankton: Long-distance dispersal of the foraminifer *Truncorotalia truncatulinoides* . Geology 36: 899–902.

[60] Williams EE (1965) The species of Hispaniolan green anoles (Sauria, Iguanidae). Breviora 227: 1–16.

[61] Peijnenburg KTCA, Goetze E (2013) High evolutionary potential of marine zooplankton. Ecol Evol 3: 2765–2781. 24567838 10.1002/ece3.644 PMC3930040

[62] Morisita M (1959) Measuring the interspecific association and similarity between communities. Mem Fac Sci Kyushu Univ, Ser E 3: 65–80.

[63] Hammer O, Harper DAT, Ryan PD (2001) PAST: Paleontological statistics software package for education and data analysis. Palaeontol Electron 4: : 9 pp.

[64] MARGO Project Members (2009) Constraints on the magnitude and patterns of ocean cooling at the Last Glacial Maximum. Nat Geosci 2: 127–132.

[65] Schlitzer R (2011) Ocean Data View. Available: http://odv.awi.de/en/home/.

