# Supplementary figures and images for: Phylogeography of the Tropical Planktonic Foraminifera Lineage Globigerinella Reveals Isolation Inconsistent with Passive Dispersal by Ocean Currents
Source: PLoS One. 2014 Mar 24;9(3):e92148. doi: 10.1371/journal.pone.0092148 (PMC3963880; doi:10.1371/journal.pone.0092148)

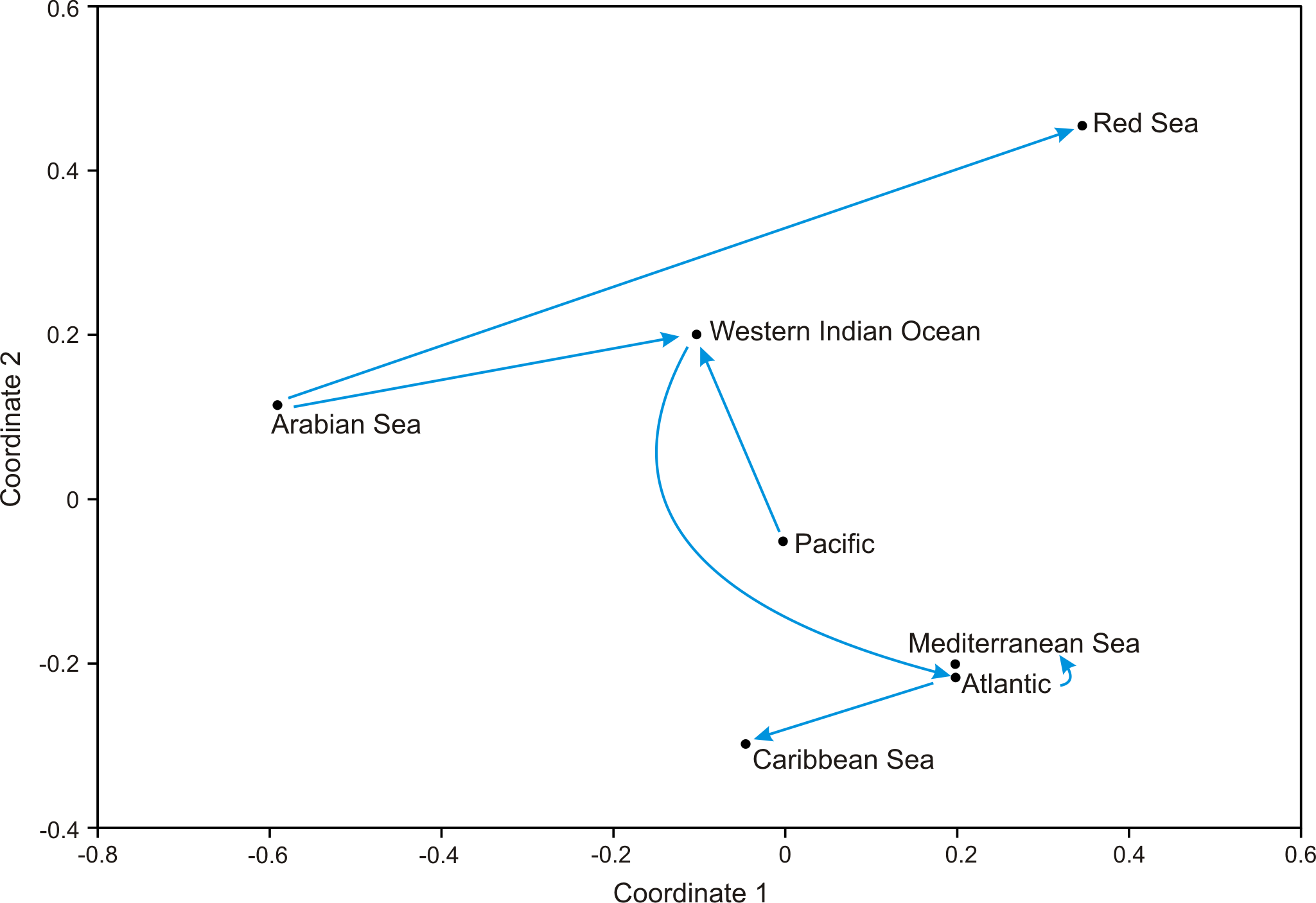

Supplement: Figure S1 — Rendition of similarity of relative abundances of all genetic types of G. siphonifera in the sampling regions. In order to statistically assess the geographical structure in the occurrence of genetic lineages of G. siphonifera, the sampling sites were separated into seven regions of the world ocean. The similarity of relative abundances of genetic lineages among these regions was visualized using non-metric multidimensional scaling based on the Morisita similarity index [62], as implemented in the PAST software v. 2. 17c [63]. Arrows indicate the direction of surface ocean currents connecting neighboring regions. (TIF) [file pone.0092148.s001.tif]
